# Editorial Comment: A Contemporary Analysis of Dual Inflatable Penile Prosthesis and Artificial Urinary Sphincter Outcomes

**DOI:** 10.1590/S1677-5538.IBJU.2020.06.07

**Published:** 2020-09-02

**Authors:** Valter Javaroni

**Affiliations:** 1 Departamento de Andrologia Hospital Federal do Andaraí Rio de Janeiro Rio de JaneiroRJ Brasil Departamento de Andrologia, Hospital Federal do Andaraí Rio de Janeiro, Rio de Janeiro, RJ, Brasil

Neal Patel ^1^, Ron Golan ^1^, Joshua A Halpern ^1^, Tianyi Sun ^2^, Abena Denise Asafu-Adjei ^3^, Bilal Chughtai ^1^, Peter Stahl ^3^, Art Sedrakyan ^2^, James A Kashanian ^1^

^1^ Department of Urology, Weill Cornell Medicine, New York, New York; ^2^ Department of Health Services and Policy Research, New York, New York; ^3^ Department of Urology, New York Presbyterian-Columbia University Medical Center, New York, New York

J Urol. 2019 Jan;201(1):141-146.

**DOI: 10.1016/j.juro.2018.07.046 | ACCESS: 10.1016/j.juro.2018.07.046**

## COMMENT

In this interesting paper Dr. Neal Patel and colleagues from New York presented an analysis of SPARCS (New York State Department of Health Statewide Planning and Research Cooperative) database for men who underwent inflatable penile prosthesis and/or artificial urinary sphincter insertion between 2000 and 2014.

Compared with men who received a penile prosthesis alone those with a penile prosthesis and an artificial urinary sphincter (not necessarily done at the same surgery) had a higher likelihood of undergoing inflatable penile prosthesis reoperation at 1 year (OR 2.08, 95% CI 1.32-3.27, p <0.01) and 3 years (OR 2.60, 95% CI 1.69-3.99, p <0.01).

The authors concluded that combined inflatable penile prosthesis and artificial urinary sphincter insertion portends a higher likelihood of inflatable penile prosthesis reoperation at 1 and 3 years. However, artificial urinary sphincter outcomes remain comparable.

These data are in opposition to 2013 publication on Journal of Urology by Dr. Segal and cols. ( 1 ) retrospectively reviewed the records of 55 combined procedures that were performed from 2000 to 2011 and concluded that dual implantation (DI) was feasible without an increased risk of adverse outcomes compared to implantation of a single prosthesis. And also contradict a 2019 publication in Urology by Dr. Boysen and cols. ( 2 ) where, with the biggest number of cases (all over 65 years old), dual implantation does not adversely affect perioperative complications or device survival relative to placement of either device alone.

One important limitation of all cited articles is the lacking on data concerning factors that could influence outcomes in prosthetic surgery such as surgeon volume, operative time, or antibiotic selection. And also the use of reoperation as a surrogate for device survival, since someone may elect not to undergo revision surgery despite mechanical failure.

The decision to treat a man with DI or staged procedure is impacted by many factors including safety and durability, but patient preference and costs are essentials issues. A small retrospective study ( 3 ) compared 15 men treated with DI and 8 treated with a staged approach. The men treated with staged device placement “would have chosen a single procedure...if asked again.” Let’s wait for more robust evidence.
